# Comparative computational and experimental analyses of some natural small molecules to restore transcriptional activation function of p53 in cancer cells harbouring wild type and p53^Ser46^ mutant

**DOI:** 10.1016/j.crstbi.2022.09.002

**Published:** 2022-09-13

**Authors:** Seyad Shefrin, Anissa Nofita Sari, Vipul Kumar, Huayue Zhang, Hazna Noor Meidinna, Sunil C. Kaul, Renu Wadhwa, Durai Sundar

**Affiliations:** a Department of Biochemical Engineering & Biotechnology, Indian Institute of Technology (IIT)-Delhi, Hauz Khas, New Delhi, 110-016, India; b AIST-INDIA DAILAB, National Institute of Advanced Industrial Science & Technology (AIST), Tsukuba, 305-8565, Japan

**Keywords:** Natural drugs, Anti-cancer drugs, p53, Ser46-mutant p53, Phosphor mutant p53, Mortalin inhibitor

## Abstract

Genetic mutations in p53 are frequently associated with many types of cancers that affect its stability and activity through multiple ways. The Ser46 residue present in the transactivation domain2 (TAD2) domain of p53 undergoes phosphorylation that blocks its degradation by MDM2 and leads to cell cycle arrest/apoptosis/necrosis upon intrinsic or extrinsic stresses. On the other hand, unphosphorylated p53 mutants escape cell arrest or death triggered by these molecular signaling axes and lead to carcinogenesis. Phosphorylation of Ser in the TAD2 domain of p53 mediates its interactions with transcription factor p62, yielding transcriptional activation of downstream pro-apoptotic genes. The p53 phosphorylation causes string-like elongated conformation that increases its binding affinity with the PH domain of p62. On the other hand, lack of phosphorylation causes helix-like motifs and low binding affinity to p62. We undertook molecular simulation analyses to investigate the potential of some natural small molecules (Withanone (Wi-N) & Withaferin-A (Wi-A) from Ashwagandha; Cucurbitacin-B (Cuc-B) from bitter Cucumber; and Caffeic acid phenethyl ester (CAPE) and Artepillin C (ARC) from honeybee propolis) to interact with p62-binding region of p53 and restore its wild-type activity. We found that Wi-N, Wi-A, and Cuc-B have the potential to restore p53-p62 interaction for phosphorylation-deficient p53 mutants. Wi-N, in particular, caused a reversal of the α-helical structure into an elongated string-like conformation similar to the wild-type p53. These data suggested the use of these natural compounds for the treatment of p53^Ser46^ mutant harbouring cancers. We also compared the efficiency of Wi-N, Wi-A, Cuc-B, CAPE, and ARC to abrogate Mortalin-p53 binding resulting in nuclear translocation and reactivation of p53 function and provide experimental evidence to the computational analysis. Taken together, the use of these small molecules for reactivation of p53 in cancer cells is suggested.

## Highlights

• Wild type p53 (p53^WT^) and its mutant form (p53^S46PΔ^) are associated with multiple cancers.
• Natural compounds serve as a potential mediator to restore the function of p53 in wild type and Ser46 phosphor mutant.
• In-silico analysis suggested that Wi-A, Wi-N, and Cuc-B are stronger inhibitors of p53 -mortalin interaction.
• These entities could also bind to p53^S46PΔ^ and mimic the phosphorylated conformation, suggesting reactivation of p53^WT^.

## Graphical abstract

**Figure:**
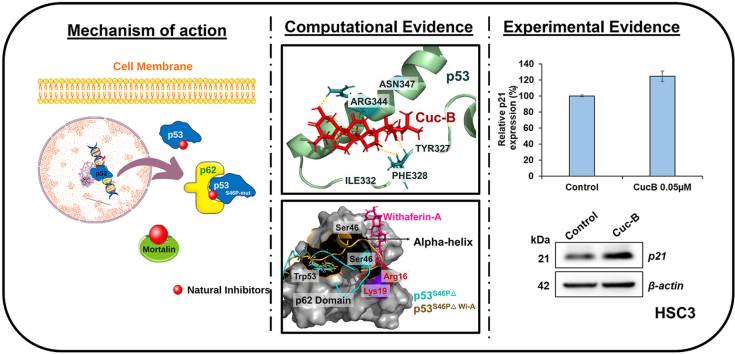

## 1 Introduction

The p53 gene, commonly known as the guardian of the genome, is a major tumor suppressor gene of which the mutations are frequently observed in large variety of cancers (Hainaut and Hollstein, 2000). The p53 protein contains four main domains, (i)Transactivation domain (TAD)- helps in initiation of transcription by binding with other transcription factors (ii) DNA binding domain-helps in identifying the damaged regions, (iii) Oligomerisation domain-helps in the formation of p53 tetramers and (iv) the regulatory domain-regulates functions of p53 in DNA damage repair, cell cycle, growth arrest or apoptosis (Balint and Vousden, 2001). Deregulation of p53 by transcription, translation, or epigenetic modifications has been reported in various types of cancers (Ashcroft et al., 1999). HDM2 is an effector and antagonist of p53 that regulates its activities through degradation by ubiquitination and proteasomal pathway. p53 gene mutations, deletions, HDM2-mediated degradation and cytoplasmic sequestration have been reported as main drivers of oncogenic signaling in a large variety of cancers (Hainaut and Hollstein, 2000; S. C. Kaul, Aida, Yaguchi, Kaur and Wadhwa, 2005; S. C. Kaul, Reddel, Mitsui and Wadhwa, 2001; Lu et al., 2011a; Unger et al., 1999).

Stress induced growth arrest/apoptosis has been shown to be mediated by post-translational modifications (phosphorylation, methylation, acetylation) of p53. Phosphorylation of the TAD domain prevents its binding to HDM2 and subsequent degradation by proteasomal pathway (Bao et al., 2001; Chehab et al., 2000; Sakaguchi et al., 2000; Tibbetts et al., 1999; Unger et al., 1999). Phosphorylation of Ser46 has been reported to be essential for binding of p53 with other cofactors like p300, p62[TFIIH], CBP, GCN5, and PC4 (Sullivan et al., 2018) and in turn is regulated by several kinases, e.g., homeodomain interacting protein kinase 2 (HIPK2), dual-specificity tyrosine-protein kinase CC δ (PKCδ), AMP-activated protein kinase catalytic subunit α (AMPKα) or p38 mitogen-activated protein kinase (p38 MAPK) (Smeenk et al., 2011). It determines the cell cycle progression, DNA damage response and apoptosis in response to intrinsic and extrinsic stresses by regulation of expression and activities of several proteins (the BCL2 family proteins-proapoptotic: BAX & BAK and antiapoptotic: BCL2, BCL-W, MCL1, BCL-XL, BIM, BAD & NOXA) (Brambilla et al., 1996). The transcription activation function of p53 requires its binding with other transcription factors, co-activators, and co-repressors. TAD consists of two sub-domains - TAD1 (residues 1–42), and TAD2 (residues 43–61) (Supplementary Fig. 1A). Whereas HDM2 and its homologs proteins bind to TAD1, TFIIH subunit p62 binds toTAD2 (Okuda and Nishimura, 2014). In the native state, the phosphorylated TAD2 forms an elongated string-like conformation with tryptophan (Trp53) buried inside deeply. In this conformation, phosphorylated p53 binds with p62 through its pleckstrin homology (PH) domain (Okuda and Nishimura, 2014). However, when the Ser46 is mutated disabling the phosphorylation at residue 46, also known as phospho-mutant, there is a significant conformational change in the string-like conformation as well as in the binding energy (Okuda and Nishimura, 2014). p53^Ser46^ phospho-mutants are commonly observed in oral squamous cell carcinoma as well as in many other cancers (Ichwan et al., 2006). Mortalin, a stress chaperone of the Hsp70 family, binds to p53, causing its sequestration in the cytoplasm. Abrogation of mortalin-p53 interaction by a variety of small molecules has been shown to yield growth arrest or apoptosis in cancer cells by reactivation of p53^wild type^ function. The interaction between the mortalin and p53 occurs at N-terminal residues (253–282) of mortalin with C-terminal residues (312–352) of p53 (Iosefson and Azem, 2010; S. C. Kaul et al., 2001). Mortalin is upregulated in large variety of cancer (Ando et al., 2014; Chen et al., 2011; Deocaris et al., 2013; Rozenberg et al., 2013). We have earlier reported several synthetic compounds like Rhodacyanine dye MKT-077, Mortaparib and Mortaparib^plus^ for their ability to intercept p53 mortalin interaction (Putri et al., 2019; A. N. Sari et al., 2021; Wadhwa et al., 2000). Furthermore, several natural metabolites such as, CAPE (from honeybee propolis), Fucoxanthin (from brown sea weeds), Wi-A and Wi-N (from Ashwagandha), Solasonine and Solamargine (from Solanum plants), Acantrifoside (*Acanthopanax trifoliatus*), Embelin (from *Embelia ribes*), Veratridine (from Veratrum and Liliaceous plants) have been shown to disrupt mortalin-p53 interaction and restore wild-type p53 activity in cancer cells (Abdullah et al., 2015; Hartati and Djauhari, 2020; Nigam et al., 2015; Pham et al., 2019).

Synthetic chemotherapeutic drugs often show excessive toxicity, adverse effects on normal body functions and drug resistance response of cancer cells evoking the use of multidrug combination for better treatment. (Housman et al., 2014). On the other hand, natural compounds either alone or in combination have been shown to possess fewer side effects and higher efficiency. Most recently, a comparative study of Wi-N, CAPE, and Wi-A showed that Wi-N and Wi-A could inhibit EGFR (Epidermal Growth Factor Receptor) and its mutant forms in the EGFR-driven lung carcinoma, while CAPE could strongly inhibit wild-type EGFR (Malik et al., 2021). Another independent study has reported that the combination of Wi-A and CAPE could inhibit mortalin-p53 interactions, and PARP1-mediated DNA repair, which led to the accumulation of DNA damage triggering apoptosis in cancer cells (Anissa Nofita Sari et al., 2020). Of note, the combination was found to be more effective than each of these molecules (Garg et al., 2020). Wi-A and CAPE were also reported to reactivate wild-type function to some p53 mutants. Wi-A, Wi-N, and the extract rich in these Withanolides caused restoration of wild-type p53 function in mutant p53^Y220C^ harboring cells associated with induction of p21^WAF−1^-mediated growth arrest/apoptosis (Sundar et al., 2019). Meanwhile, CAPE was also shown to restore the structurally unstable mutant of p53, p53^Y220C^ (Radhakrishnan et al., 2021). In light of these premises, we have investigated the comparative efficiency of these natural compounds to reactive Ser46 mutant of p53 by computational approaches. We found that some of these compounds like Wi-N or Wi-A when used in combination with Cuc-B may offer multi-modal ability to restore wild-type p53 activity. These include, abrogating p53 mortalin interaction and formation of the elongated string-like conformation of TAD causing an increase in the binding affinity of p53 with the transcription factor p62. Such dual effects could be beneficial for cancer treatment.

## 2 Methodology

### 2.1 Pre-processing and preparation of target protein as well as ligands

The NMR structure of the TAD2 domain of p53 bound with p62 [PDB ID; 2RUK] was downloaded from the RCSB protein data bank (Okuda and Nishimura, 2014). The initial structure of the p53 bound p62 was imported into Maestro version 2018–3 of Schrodinger software. The phosphorylated ser46 residue on p53 was mutated to normal ser46 to create phospho-mutant p53. For abrogation of Mortalin-p53 interactions, we used chain-A of p53 tetramerization domain [PDB ID 4MZR] and the complete structure of Mortalin [PDB ID:4KBO] (Amick et al., 2014; Papeo et al., 2015). Using the protein preparation wizard of the maestro, pre-processing such as the addition of missing disulfide bonds, conversion of selenomethionines to methionines, deletion of all the water molecules, missing hydrogen atoms were added, terminals were capped, and the missing side chains and loops were checked (none found) (Bowers et al., 2006; Sastry et al., 2013). Using the review and modification option of the protein preparation wizard, all the co-crystallized hetero atoms from the target structure and hydrogen bonds were optimized. Finally, applying OPLS3e forcefield with pH range 7.0, restrained minimization was done until the average Root Mean Square Deviation (RMSD) of the non-hydrogen atoms converged to 0.30 ​Å. The phosphorylated wild-type p53 bound p62 as well as the phospho-mutant p53 bound p62 were simulated for 200ns separately without any ligands to get the converged structure for references. The structure of Cucurbitacin-B (ID:5281316), Wi-N (ID:2169027), Wi-A(ID:265237), CAPE (ID:5281787) and, Artepillin-C [ARC] (ID:444637) were downloaded from the PubChem database (https://pubchem.ncbi.nlm.nih.gov/). The ligands and proteins along with the accession IDs used are shown in Table 1. These ligands were then prepared for docking and simulation studies using the LigPrep module of the Schrodinger suite (Sastry et al., 2013). The Ligand preparation steps involved the minimization using the OPLS3e force field and the generation of the possible ionization sates at PH 7.0 ​± ​2.0. (Greenwood et al., 2010). The ligands were subsequently desalted and allowed to generate tautomers. In the stereoisomer's tab of the Ligprep tool, the Retain option specified chiralities and allowed to generate a maximum of 32 per ligand. The low energy ring conformation was generated one per ligand. (Sastry et al., 2013).

**Table 1:** Summary of the structures downloaded from PDB and PubChem along with their identification number.

SLN0	NAME	IDENTIFICATION NUMBER
1	p53-p62 complex	PDB id: 2RUK
2	Cucurbitacin-B (Cuc-B)	PUBCHEM ID:5281316
3	Withanone (Wi-N)	PUBCHEM ID:2169027
4	Withaferin-A (Wi-A)	PUBCHEM ID:265237
5	Caffeic acid phenethyl ester (CAPE)	PUBCHEM ID:5281787
6	Artepillin C (ARC)	PUBCHEM ID:444637

### 2.2 HADDOCK 2.2 protein-protein docking server

To identify the interacting residues and the type of interactions between p53 and mortalin, the structures obtained from PDB for p53 and Mortalin were docked specifying a range of residue which has been earlier reported using the HADDOCK2.2 online server(van Zundert et al., 2016). The earlier reported binding site of p53 to mortalin was assigned to amino acid residues 323–337.Mortalin amino acid residues 253–282 were reported to interact with p53(Iosefson and Azem, 2010; S. C. Kaul et al., 2001). The structure from the cluster, which had highest HADDOCK score with maximum binding energy with more negative value and lowest RMSD change was downloaded and further simulated to get a converged structure.

### 2.3 Molecular docking of ligands

The glide module of the Schrodinger suite was used for all the docking studies using the OPLS3e force field. The grid for docking was generated on the ser46 residue of phospho-mutant p53 in the TAD2 domain. The average structure obtained from the converged simulation of phosphor deficient p53/p62 complex was used as the target protein for docking. The grid file was further used to dock Cucurbitacin-B, Wi-A, Wi-N, CAPE, and ARC ligands using glide extra precision flexible docking (Friesner et al., 2006). For p53 mortalin abrogation, the grid generated for p53 were along the residues Tyr327, Gln331 and Arg333. The interacting residue Glu263 has been taken as the central residue for generating the grid for Mortalin.

### 2.4 MD simulation in water

Desmond package in the maestro from the Schrodinger suite was used to study the stability of the protein-ligand system (Bowers et al., 2006). Firstly, systems were built using the system builder program of Desmond using the OPLS3e force field; for solvation, a predefined TIP4P water model was chosen. In the boundary conditions option, an orthorhombic periodic boundary was set up to give the shape and size of the box buffered at a distance of 10 ​Å, and then ions were added to every system for balancing the charge. After building the solvated protein-ligand complex systems, the energy of the prepared systems was minimized by running 100 ps low-temperature (10K) Brownian motion MD simulation (NVT ensemble) to remove steric clashes. (Harder et al., 2016). Further, the minimized systems were equilibrated in seven steps in NVT and NPT ensembles using the “relax model system before simulation” option in the Desmond Schrodinger suite. Finally, Molecular dynamic simulations were performed with the periodic boundary condition in the NPT ensemble. The pressure and temperature of the systems were kept at 1 atmospheric pressure (using Martyna–Tobias–Kelin barostat) and 300 ​K temperature (using Nose–Hoover chain thermostat), respectively. The production run of 200 ​ns was performed while saving the configuration at every 200 ​ps interval.

### 2.5 Analysis of MD trajectory

The MD trajectories were analyzed post molecular dynamics simulations using the Desmond Simulation event analysis tool (Bowers et al., 2006). Root Mean Square Deviation (RMSD) of protein-natural compound complexes were analyzed as a function of time to investigate the stability of ligands in the phosphor mutant Ser46 region. The number of hydrogen bonds between the ligands and protein throughout the simulation time was calculated using default parameters. Then, the Desmond simulation interaction diagram tool was used to analyze the residues making contacts throughout the simulation and their occupancy percentage. The radius of gyration, Solvent accessible surface area, RMSD of ligands have also been calculated and compared to investigate their flexibility, binding inside the active pocket of protein, and their stability throughout the simulation (Liu and Kokubo, 2017).

### 2.6 MM/GBSA free energy calculations

The representative structures obtained from the simulation have been split into chains to identify binding energy between the TAD2 domain of p53 and the PH domain of p62. Post MD, from the trajectories, a total of 100 structures were extracted from the duration of 0 ​ns–200 ​ns of the simulations time. The average structure obtained from these 100 extracted structure complexes was used to calculate the MM/GBSA free binding energy using the ‘prime MM-GBSA’ of the Maestro Schrodinger suite(Li et al., 2011).

The equation used for the calculation was:

MM/GBSA ΔG _bind__=_ΔG _complex_^__^ (ΔG _receptor_ ​+ ​ΔG _ligand_)

ΔG ​= ​ΔE_Gas_ ​+ ​ΔG_Sol_ – TΔS_Gas_

ΔE_Gas_ ​= ​ΔE_Int_ +ΔE_Ele_ ​+ ​ΔE_vdw_

ΔG_Sol_ ​= ​ΔG_gb_ ​+ ​ΔG_Sur_

ΔG _complex_, ΔG _receptor,_ and ΔG _ligand_ represent the free energies of the complex, receptor, and ligand, respectively. MM/GBSA refers to the binding affinity of the ligand towards the target protein; a more negative value represents stronger affinity (Genheden and Ryde, 2015). The binding energy calculated is not absolute, it's the relative energy of the apo-complex and docked complex. This is because of limitations in the force field and ignorance of entropy terms in the algorithm. The binding free energy (ΔG_bind_) was dissociated into the binding free energy of the complex, receptor, and ligand. The gas-phase interaction energy (ΔE_gas_) was calculated as the sum of electrostatic (ΔE_elec_) and van der Waal (ΔE_vdw_) interaction energies, while internal energy was neglected. The solvation free energy (ΔG_sol_) contains non-polar (ΔG_surf_) and polar solvation energy (ΔG_gb_), which was calculated using the VSGB solvation model and OPL3e force field. The entropy change -TΔS upon ligand binding is often neglected in the calculation due to time expense. However, higher binding energy indicates a higher binding affinity.

### 2.7 Cell culture and reagent

Human osteosarcoma (U2OS; wild type p53) and oral squamous carcinoma (HSC3; p53^Ser46mutant^) cells were obtained from the Japanese Collection of Research Bioresources (JCRB, Tokyo, Japan). They were cultured in Dulbecco's Modified Eagle's Medium (DMEM) (Invitrogen, Carlsbad, CA, USA) supplemented with 5–10% fetal bovine serum (Fujifilm WAKO Pure Chemical Corporation, Osaka, Japan), 1% penicillin-streptomycin at 37 ​°C in an atmosphere of 5% CO2.

### 2.8 Drug preparation and treatments

The stock solutions (5 ​mM) of Artepillin C, CAPE, Cucurbitacin-B, Withaferin-A and Withanone were prepared by dissolving in Dimethyl Sulfoxide (DMSO) (WAKO, Osaka, Japan). Each of them was diluted in complete cell culture media to give working concentrations of 300 ​μM (Artepillin C), 20 ​μM (CAPE), 5 ​μM (Cucurbitacin-B), 4 ​μM (Withaferin-A), and 50 ​μM (Withanone). Treatment of Wi-A, CAPE, or their combination was performed in the cells with 60–70% of confluency for 24–48 ​h.

### 2.9 Western blotting

Control and treated cells were harvested and lysed using RIPA Lysis Buffer (Thermo Fisher Scientific, Waltham, MA, USA) with complete protease inhibitor cocktail (Roche Applied Science, Mannheim, Germany) in it. They were vortexed at 4 ​°C for 30 ​min. Lysates were centrifuged at 15,000 ​rpm for 10 ​min. The supernatant was subjected to BCA protein assay (Thermo Fisher Scientific, Waltham, MA, USA) to determine the protein concentration of each sample followed by Western blotting. The cell lysates (10–20 ​μg) were separated in 10% SDS-polyacrylamide gel electrophoresis (SDS-PAGE), then transferred to a polyvinylidene difluoride (PVDF) membrane (Millipore, Billerica, MA, USA) using a wet transfer [Tank Blotting Cells system, Mini Trans-Blot® Cell] (BIO-RAD, California, USA). Membrane blocking was done using 3% of bovine serum albumin at room temperature for 1 ​h. Blocked membranes were probed with the target protein-specific primary antibodies overnight at 4 ​°C. The primary antibodies used were p21^WAF1/Cip1^ (12D1) (Cell Signaling Technology) and p53 (DO-1): sc-126 (Santa Cruz Biotechnology, CA, USA). The blots were then incubated with horseradish peroxidase (HRP)-conjugated secondary antibodies (anti-rabbit IgG or anti-mouse IgG (Santa Cruz Biotechnology, CA, USA)) and developed using the enhanced chemiluminescence system (GE Healthcare, Buckinghamshire, UK). Direct-BlotTM HRP anti-β-actin antibody (Lot303080) (BioLegend CNS, Inc, San Diego, California, United States) was used as an internal control. The protein band images were analyzed by ImageJ (National Institutes of Health, Bethesda, MD, USA) software.

### 2.10 Immunocytochemistry

U2OS cells (4 ​× ​10^4^ per well) were plated on 18-mm glass coverslips placed in 12-well plates and allowed to settle overnight. After 24 ​h, the cells were treated with Artepillin C, CAPE, Cucurbitacin-B, Withaferin-A and Withanone for 24 ​h, then washed twice with PBS and fixed in methanol:acetone (1:1) at 4 ​°C for 5–10 ​min. Cells were washed with PBS. Permeabilization of the cells was performed using PBS with 0.1% Triton X-100 (PBST) for 10 ​min, followed by blocking using 2% of bovine serum albumin in PBST at room temperature for 1 ​h. Fixed cells were incubated with primary antibodies diluted in 2% of bovine serum albumin in PBST. The primary antibodies used were p21^WAF1/Cip1^ (12D1) (Cell Signaling Technology) and p53 (FL-393) (sc-6243) (Santa Cruz Biotechnology, CA, USA). Immunostaining was visualized by 1–2 ​h of incubation with secondary antibody staining (Texas RED (Amersham Biosciences, Buckinghamshire, UK) or fluorescein isothiocyanate (FITC), and Alexa-488 or Alexa-594 conjugated antibodies (Molecular Probes, Eugene, OR, USA)). Hoechst 33342 (Invitrogen, Molecular Probes, Eugene, OR, USA) was used for nuclear staining. The coverslips were mounted on glass slides and examined under Zeiss Axiovert 200 ​M microscope (40 ​× ​objective lens) with AxioVision 4.6 software (Carl Zeiss, Tokyo, Japan). Protein expression represented by the fluorescence signals was quantified using ImageJ software (National Institutes of Health, Bethesda, MD, USA).

### 2.11 Luciferase reporter assay

U2OS cells stably transfected with luciferase reporter driven by p53-responsive promotor (PG13) were seeded (1 ​× ​10^5^/well) in 6-well plate and allowed to adhere at 37 ​°C in a humidified CO_2_ incubator overnight. The cells were treated with either DMSO (0.05% - control), Artepillin C, CAPE, Cucurbitacin-B, Withaferin-A or Withanone for 24 ​h. The cells were then washed with PBS, collected and lysed using the passive lysis buffer (PLB) (Promega, WI, USA). Protein lysates were subjected to quantification using bicinchonic acid assay (BCA) (Thermofisher Scientific, Rockford, IL). Luciferase activity was measured using Dual-Luciferase® Reporter Assay System (Promega, Japan) following the manufacturer's instructions.

### 2.12 Statistical analysis

Statistical data from three or more independent experiments were expressed as mean ​± ​standard deviation. Unpaired *t*-test (GraphPad Prism, GraphPad Software, San Diego, CA) was performed to determine statistical significance between the control and experimental samples. Values of p ​> ​0.05 (ns), p ​≤ ​0.05 (∗), p ​≤ ​0.01 (∗∗), p ≤ 0.001 (∗∗∗), and p ​≤ ​0.0001 (∗∗∗∗) were considered statistically non-significant, significant, very significant, highly significant, and extremely significant, respectively.

## 3 Results

The structure obtained for the p53 with phosphorylated Ser46 was mutated to unphosphorylated Ser46 and simulated for 200ns to get the converged structure. After preparing the protein and ligands using protein preparation Wizard and Liprep module respectively, a grid was generated for docking taking Ser 46 residue as the center of the grid. The test compounds obtained from the PubChem database were docked against the generated grid and further simulated for 200ns. Docking scores for all the ligands were recorded and shown in the **(**Table 2**)**.

**Table 2:** Docking scores of the ligands which bound with mutated p53/p62 complexes.

SLN0	NAME	DOCK SCORE
1	Cucurbitacin-B (Cuc-B)	-3.710
2	Withanone (Wi-N)	-3.976
3	Withaferin-A (Wi-A)	-2.636
4	Caffeic acid phenethyl ester (CAPE)	-4.156
5	Artepillin C (ARC)	-0.771

### 3.1 Structural variation between the interactions of wild-type p53 and phospho-mutant p53 with p62

**Fig. 1:**
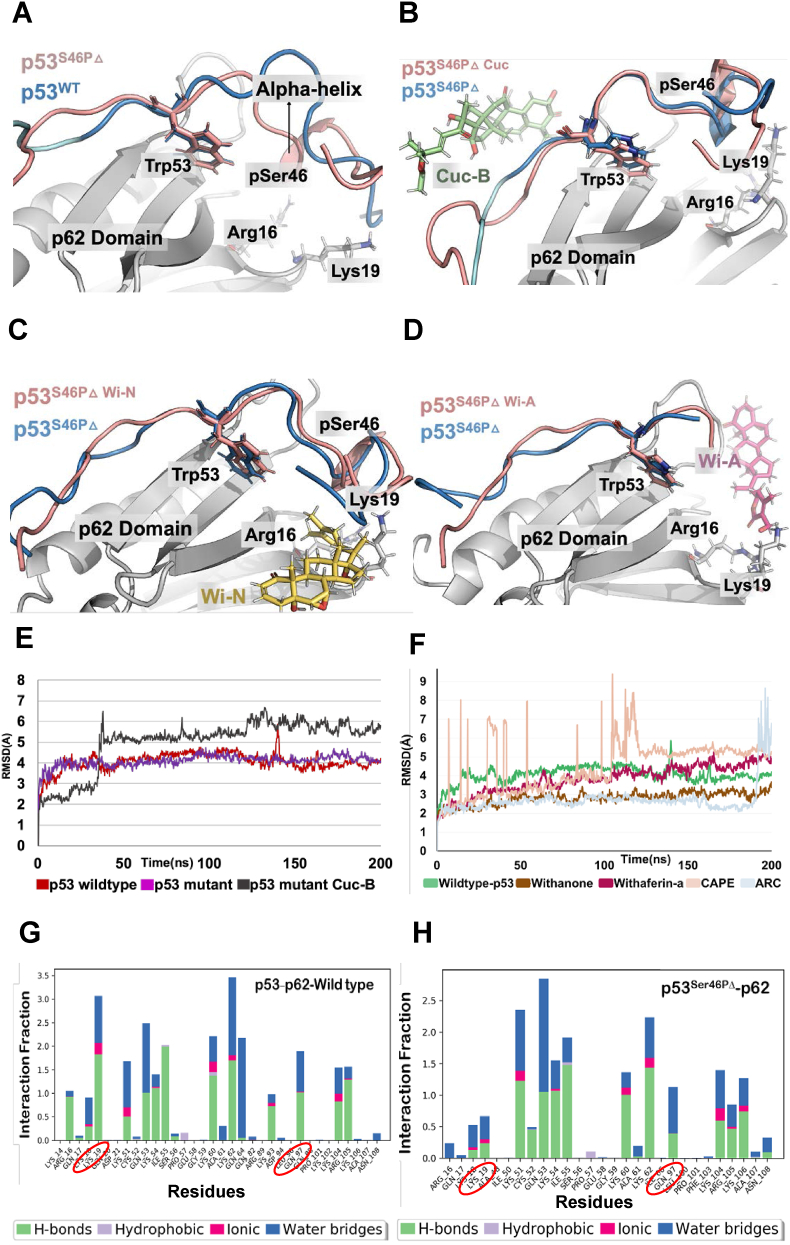
Molecular dynamic simulation study of natural metabolites on phosphor mutant p53-p62 complex. A) Conformational changes observed in the structure of mutant type p53 compared with wild-type p53. B) Conformational changes observed in the structure of mutant type p53 after Cuc-B interaction. C) Conformational changes observed in the structure of mutant type p53 after Wi-N interaction. D) Conformational changes observed in the structure of mutant type p53 after Wi-A interaction. E) Root mean square deviation plot of wild-type as well as mutant p53 complex with p62 before and after Cuc-B interaction simulated for 200ns. F) Root mean square deviation plot of mutant p53 complex with p62 before and after Wi-N, Withaferin-A, CAPE and ARC interaction simulated for 200ns. G) Interaction fraction diagram of wild type p53 with amino acids of p62. H) Interaction fraction diagram of phospho-mutant p53 with amino acids of p62.

**Fig. 2:**
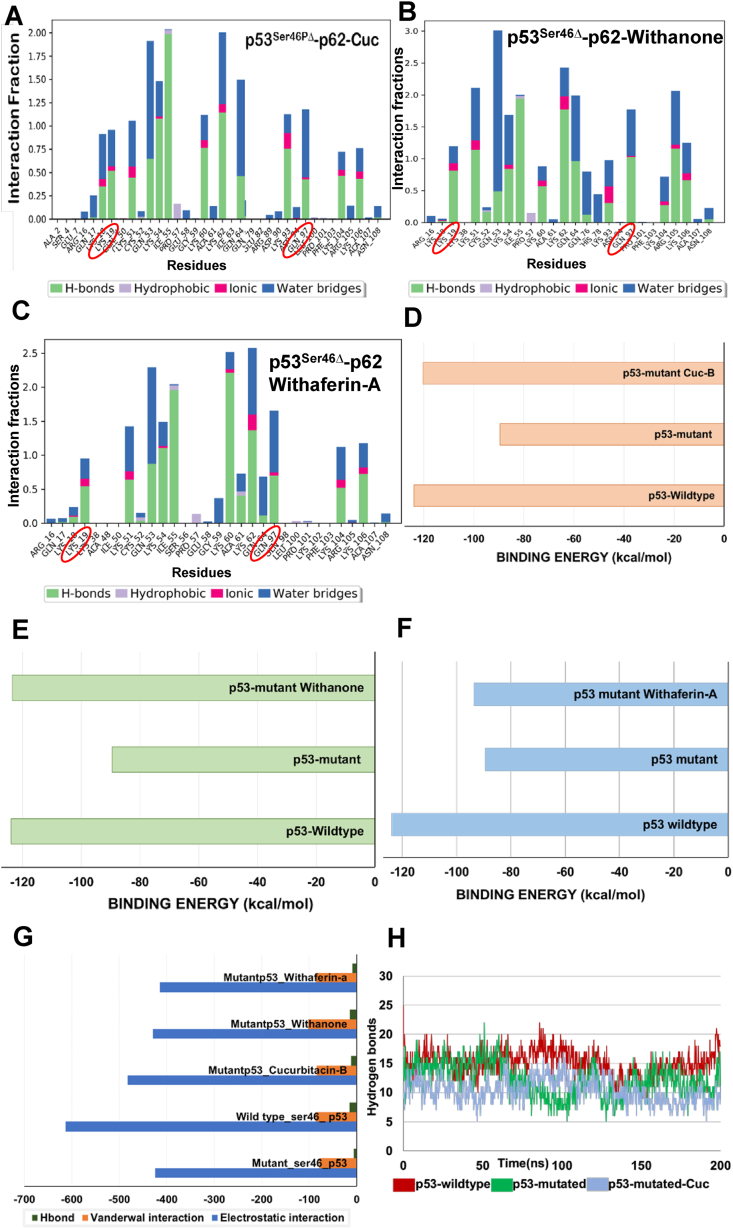
Molecular dynamics simulation analysis of natural metabolites in restoring wild type p53 activity. A) Interaction fraction diagram of phospho-mutant p53 with amino acids of p62 after Cuc-B intervention. B) Interaction fraction diagram of phospho-mutant p53 with amino acids of p62 after Wi-N intervention. C) Interaction fraction diagram of phospho-mutant p53 with amino acids of p62 after Wi-A intervention. D) Change in MMMGBSA binding energy before and after Cuc-B interaction. E) Change in MMMGBSA binding energy before and after the Wi-N interaction. F) Change in MMMGBSA binding energy before and after the Wi-A interaction. G) Change in Vander Wal, electrostatic and hydrogen bonding energy of interacting molecules. H) Hydrogen bonding plot observed between p53 and p62 before and after Cuc-B intervention.

The NMR complex of wild-type p53 chain interacting with PH domain of p62 obtained from PDB was mutated to remove the phosphorylation at Ser46 residue. The phospho-deficient mutant obtained after the mutation on Ser46 amino acid was further simulated for 200ns to get a converged conformation for the mutant p53. The protein complex was visualized using pymol and found that the structure of phospho-deficient p53mutant showed a small alpha-helical domain near the N terminal region of p53, while in the wild-type p53 this alpha-helical domain was not observed (Fig. 1A). An elongated string-like conformation of Ser46, Thr55 phosphorylated p53 on the p62 domain was also reported earlier in a previous study which is absent in the phosphor-mutant p53 (Okuda and Nishimura, 2014). The structures used for the comparison were the average structures obtained from converged 200 ns simulation performed in the TIP4P water model, which is evident from the stabilized Root mean square deviation plot obtained for both wild-type and phosphor-mutant p53 complex with p62 (Fig. 1E). The simulation interaction diagram suite of Schrodinger showed that there is a significant decrease in the hydrogen bonding when the phospho-deficient mutant p53 was complexed with p62 residues, namely, Arg16, Lys19, and Gln97 when compared with the wild-type p53 (Fig. 1G and H). Further, after analysing the average structure obtained from the simulation we observed slight detachment of the Ser46 phosphor-mutant p53 from the p62 causing a slight opening of the complex. The mutation at Ser46 residue on p53 can be a potential reason for this loss of hydrogen bonding in Arg16, Lys19, and Gln97. The hydrogen bonds formed between p53 and p62 of both wild-type p53 and phospho-deficient mutant p53 were analyzed and observed a decline of hydrogen bonds in mutant p53 which further supported our data (Fig. 2H).

### **3.2** **Interaction of natural metabolites on p53**^**S46P**Δ^**- p62 complex**

Initially, we examined docking of CAPE and ARC on p53 wild type (p53^WT^) and Ser46 phospho-deficient mutant (p53^S46PΔ^) and found that both of these docked well with the grid generated along with the phosphor-deficient mutant residue with a docking score of −4.156 kcal/mol-0.771 ​kcal/mol, respectively (Table 1). However, the docked ligands were not stable at the binding pocket and the root means square deviation plot showed higher deviation that implied unstable binding in the active region (Fig. 1F). Next, Cuc-B was docked with the p53^WT^ and p53^S46PΔ^ mutants. The average protein complex obtained from the simulation was visualized using pymol and found that, the Cuc-B showed binding at an allosteric site away from the Ser46 residue (Fig. 1B). Further, the elongated string-like conformation found in the wild-type p53 is not formed due to the interaction with Cuc-B**.** It showed stable interaction with mutant p53 residue during simulation for 200ns as shown in the Root Mean Square Deviation plot (Fig. 1E). The MMGBSA binding energy between the p53^S46PΔ^ and p62, showed decrease (from −123.94 ​kcal/mol for p53^WT^ to −89.53 ​kcal/mol for p53^S46PΔ^). Of note, the MMGBSA binding energy showed increase (−120.29 ​kcal/mol) upon interaction with the Cuc-B (Fig. 2D). The increase in the binding energy of p53^S46PΔ^-p62 was further supported by the increase in hydrogen bonding at Lys19 and Gln97 from the interaction fraction diagram obtained for the 200ns simulation (Fig. 2A). The position of Trp53, which is also considered as a crucial factor in determining the binding affinity with p62, remained unchanged in Cuc-B interaction. Unlike, Cuc-B, Wi-N interacted within the mutated Ser46 residue after molecular docking and simulation. The average protein complex obtained from the simulation were visualized using pymol and found that, Wi-N showed binding near the mutated Ser46 residue, and moreover, it caused the reformation of the elongated string-like conformation found in the wild-type p53 (Fig. 1C). Wi-N showed stable interaction with phospho-deficient mutant p53 residue during the simulation of 200ns, as observed in the Root Mean Square Deviation plot (Fig. 1F). The MMGBSA binding energy between the p53^S46PΔ^ and p62, which was reduced due to mutation from −123.94 ​kcal/mol to −89.53 ​kcal/mol, was found to increase to −123.49 ​kcal/mol upon interaction with the Wi-N molecule (Fig. 2E). The increase in the binding energy was further supported by the increase in hydrogen bonding at Lys19 and Gln97 on the interaction fraction diagram obtained for the 200ns simulation (Fig. 2B). The absence of a small alpha-helical structure near the N terminal of p53 confirmed the ability of Wi-N to re-establish wild-type activity in mutant p53. However, the position of Trp53, which is considered as a crucial factor in increasing the binding affinity with p62, remained unchanged during the entire simulation length. Thus, Wi-N was found to be better entity compared to Cuc-B for binding within the region of Ser46 mutated amino acid and helped in the formation of elongated string conformation of p53 on the p62 causing increase in binding energy. Similar to Wi-N, Wi-A also showed binding within the region of Ser46 mutation after docking and simulation of the complex. The average protein complex obtained from the simulation were visualized using pymol and found that, Wi A showed binding near the mutated Ser46 residue. The elongated string-like conformation was not found in the Wi-A bound phospho-deficient p53 mutant, unlike Wi-N bound or the wild-type p53 (Fig. 1D). Wi-A showed a stable interaction with phospho-deficient mutant p53 residue during the simulation of 200ns, which is depicted in the Root Mean Square Deviation plot (Fig. 1F). The position of Trp53 remained unchanged during the entire simulation length which increases the binding affinity with p62.The MMGBSA binding energy between the mutant p53 and p62, which has been reduced due to mutation from −123.94 ​kcal/mol to −89.53 ​kcal/mol, was increased to −93.73 ​kcal/mol on interaction with Wi-A (Fig. 2F). The increase in the binding energy was further supported by the increase in hydrogen bonding at Lys19 and Gln97 on the interaction fraction diagram obtained for the 200ns simulation. (Fig. 2C).

There was a significant decrease in the electrostatic energy and hydrogen bond interaction upon the change of wild-type p53 to the mutant p53 (Fig. 2G). This was attributed to the reduction in the hydrogen bonds formed by residues like Lys19 and Gln97 which caused the entanglement of elongated string in Ser46 mutated p53. This loss of hydrogen bond can also be the reason for the decrease in the MMGBSA binding energy. The Van der Waals energy remained unchanged for wild-type p53 and mutant p53. However, the reduction in the electrostatic and hydrogen bonding energy in drug-bound complex were remarkable. For example, Wi-N caused an increase in the overall binding energy by increasing the Van der Waals forces and had no effect on electrostatic or hydrogen bond energy (Fig. 2G). There was a slight increase in the hydrogen bonding energy, which could compensate for the reduction of electrostatic energy in overall binding energy between the p53 and p62. From these results, it was evident that both Wi-N and Wi-A could increase the binding energy of p53 with p62 and both the compounds can restore wild-type p53 activity in Ser46 phospho-mutant, at least in part.

### 3.3 Effect of natural metabolites on p53-mortalin binding

**Fig. 3:**
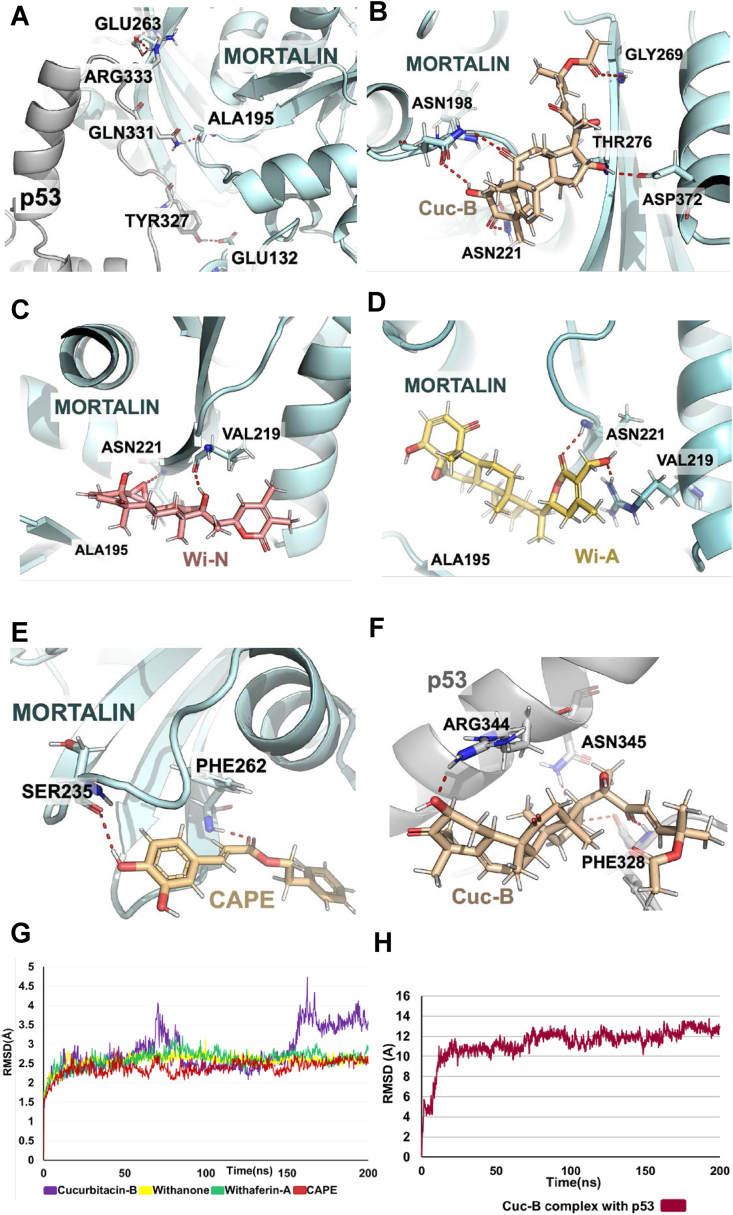
Molecular dynamics simulation study of Mortalin p53 abrogation by natural metabolites. A) Three-dimensional visualization of interaction between Mortalin and p53 (docked using HADDOCK server and simulated for 200ns). B) Three-dimensional visualization of Interaction between Cuc-B and p53 binding domain of Mortalin. C) Three-dimensional visualization of Interaction between Wi-N and p53 binding domain of Mortalin. D) Three-dimensional visualization of interaction between Wi-A and p53 binding domain of Mortalin. E) Three-dimensional visualization of interaction between CAPE and p53 binding domain of Mortalin. F) Three-dimensional visualization of Interaction between Cuc-B and Mortalin binding domain of p53. G) Root mean square deviation plot of natural ligands bound Mortalin complex showing stable interactions. H) Root mean square deviation plot of Cuc-B bound p53 complex showing stable interaction.

For analysis of the capability of the compounds for abrogation of p53-Mortalin interaction, protein-protein docking was done using HADDOCK using interacting residues between the p53 and Mortalin (van Zundert et al., 2016). The amino acid residues Tyr327, Gln331, and Arg333 of p53 formed hydrogen bonds with Glu132, Ala195, and Glu263 (Fig. 3A). Separate grids were generated, on p53 by choosing residues Tyr327, Gln331, and Arg333, and on Mortalin by selecting Glu263 as the central residue. Natural metabolites obtained from the PubChem database were docked with both the grid generated and further simulated for 200ns. Docking score and ligand binding energy (MMGBSA) are shown for all the ligands interacting with p53 and Mortalin shown in Table 3. Amino acids that interact with the ligands are summarized in Table 4.

**Table 3:** Dock scores and MMGBSA binding energy for the interaction of the ligands with p53 and Mortalin.

Natural metabolites	Wi-N		Wi-A		Cuc-B		CAPE		ARC	
Interactions	Mortalin	p53	Mortalin	p53	Mortalin	p53	Mortalin	p53	Mortalin	p53
Docking score	-2.623	-4.30	-2.792	-2.30	-5.541	-4.604	-3.202	-3.364	0.110	-0.886
MMGBSA Binding energy	-75.43 ​kcal/mol	No Stable Interaction	-80.57 Kcal/mol	No Stable Interaction	-55.52 Kcal/mol	-59.56 Kcal/mol	-51.57 Kcal/mol	No Stable Interaction	-43.12 Kcal/mol	-53.82 Kcal/mol

**Table 4:** Interacting residues of p53 and Mortalin with the ligands after simulation for 200ns.

Natural metabolites	Wi-N		Wi-A		Cuc-B		CAPE		ARC	
Interactions	Mortalin	p53	Mortalin	p53	Mortalin	p53	Mortalin	p53	Mortalin	p53
Hydrogen Bonds	Val219 Asn221	No Stable Interactions	Arg202 Val219 Arg218	No Stable Interactions	Asn198 Asn221 Thr267 Asn268	Phe328 Tyr327 Arg344	Phe262 Ser235	No Stable Interactions	No Stable Interactions	Asn345
Hydrophobic interactions	Arg218 Ile220 Thr224 Lys206	No Stable Interactions	Phe262 Leu228 Gln424 Val427 Leu428	Ile463 Gly464 Trp531	Thr270 Ser266 Tyr196 Val264	Tyr327 Asn345 Arg344 Phe341	Gln424 Thr224 Leu232 Asp233 Glu236		Val427 Ile423 Met70 Gly72 Lys73 Gln74	Phe328 Phe338 Leu330 Phe341

Molecular docking analysis revealed that Cuc-B could bind with Mortalin at its p53 binding domain (dock score ​= ​−5.54) (Table 3). Average structure extracted from the simulation was visualized using pymol and found that, Cuc-B showed binding within the cavity of the p53-binding region of mortalin domain forming key interactions with Asn221, Ser266, Thr267, and Asp268 (Fig. 3B). The MMGBSA binding energy of Cuc-B with Mortalin was found to be −55.52 ​kcal/mol (Table 3). There was a minor deviation in the RMSD plot (Fig. 3G) that could be due to the bending of the long tail of Cuc-B, which is evident from the frames extracted from 100 ns to 200 ns with an interval of 10 ns (Supplementary Fig. 2E). Further, Cuc-B docked into the mortalin-binding domain of p53 from residues 323 to 337 (dock score ​= ​-4.60 ​kcal/mol) (Table 3). The average structure obtained from the trajectory of 200 ns simulation showed that the interactions with p53 on key residues Arg344, Asn345, Tyr327, and Phe328 were involved in the mortalin-binding (Fig. 3F). The stable RMSD plot and hydrogen bond plot confirms the strong Cuc-p53 binding throughout the simulation (Fig. 3H) (Supplementary Fig. 1B). The MMGBSA binding energy of Cuc-B with p53 was found to be −59.56 ​kcal/mol (Table 3). Molecular docking analysis revealed that Wi-N could bind near the p53 binding region of Mortalin (dock score ​= ​−2.623) (Table 3). The average structure extracted from converged simulation frames shows that Wi-N binds very close to Ala195 which is a key residue in p53-Mortalin complex formation (Fig. 3C). The RMSD plot and hydrogen bond plot for the 200ns simulation revealed that the interaction is stable and forming hydrogen bonds throughout the simulation (Fig. 3G) (Supplementary Fig. 1B). The interaction fraction diagram shows that the residues Val219 and Asn221 form hydrogen bonds during a maximum period in the simulation (Supplementary Fig. 2A). The MMGBSA binding energy of Wi-N with Mortalin was found to be -75.43 ​kcal/mol (Table 3). However, Wi-N was not able to bind in the Mortalin binding region of p53. Molecular docking analysis revealed that Wi-A could bind to p53 near to its binding region of Mortalin (dock score ​= ​−2.792) (Table 3). The average structure extracted from converged simulation frames shows that Wi-A binds very close to Ala195, which is a key residue in p53-Mortalin complex formation (Fig. 3D). The RMSD plot and hydrogen bond plot for the 200ns simulation revealed that the interaction was stable and retained hydrogen bond interaction throughout the simulation (Fig. 3G, Supplementary Fig. 1B). The interaction fraction diagram showed that the residues Val219 and Arg218 formed hydrogen bonds during a maximum period in the simulation (Supplementary Fig. 2C). The MMGBSA binding energy of Wi-A with Mortalin was found to be -80.57 ​kcal/mol (Table 3). However, Wi-A did not form a stable interaction with the Mortalin binding region of p53. Molecular docking analysis revealed the ability of CAPE to bind to the p53 binding region of Mortalin (dock score ​= ​−3.202) (Table 3). The average structure extracted from converged simulation frames showed that it interacts with Phe262 which lies within the p53 binding domain of Mortalin (Fig. 3E). The RMSD plot and hydrogen bond plot for the 200ns simulation revealed that the interaction is stable and, forming hydrogen bonds throughout the simulation (Fig. 3G, Supplementary Fig. 1B). The interaction fraction diagram showed that the residues Phe262, Ser235, and Thr224 formed hydrogen bonds during a maximum period in the simulation (Supplementary Fig. 2B). The MMGBSA binding energy of CAPE with Mortalin was found to be -51.57 ​kcal/mol (Table 3). However, CAPE did not form stable interaction with the Mortalin binding region of p53. The average structure extracted from converged simulation frames although showed that ARC could not bind to the p53 binding domain of Mortalin (Supplementary Fig. 1D), the RMSD plot for the 200ns simulation revealed that the interaction was stable forming hydrogen bonds throughout the simulation (Supplementary Fig. 1F). However, ARC could be able to bind firmly within the Mortalin binding region of p53 (Supplementary Fig. 1E). The RMSD plot of the 200ns simulation revealed that the interaction is stable throughout the simulation (Supplementary Fig. 1G). The interaction fraction diagram shows that the residues Phe328 and Phe338 form hydrophobic interactions during a maximum period in the simulation (Supplementary Fig. 2D). The MMGBSA binding energy of ARC with p53 was found to be −53.582 ​kcal/mol (Table 3). These data revealed that amongst the tested compounds, only Cuc-B could interact with both p53 as well as mortalin binding regions. Two withanolides, Wi-N and Wi-A, which were found to be a good candidate for restoration of wild type activity in p53^S46PΔ^ mutant, showed strong binding with the p53-binding region of mortalin. CAPE and ARC showed relatively weaker potential.

### 3.4 Experimental evidence to the effect of natural metabolites on reactivation of p53

**Fig. 4:**
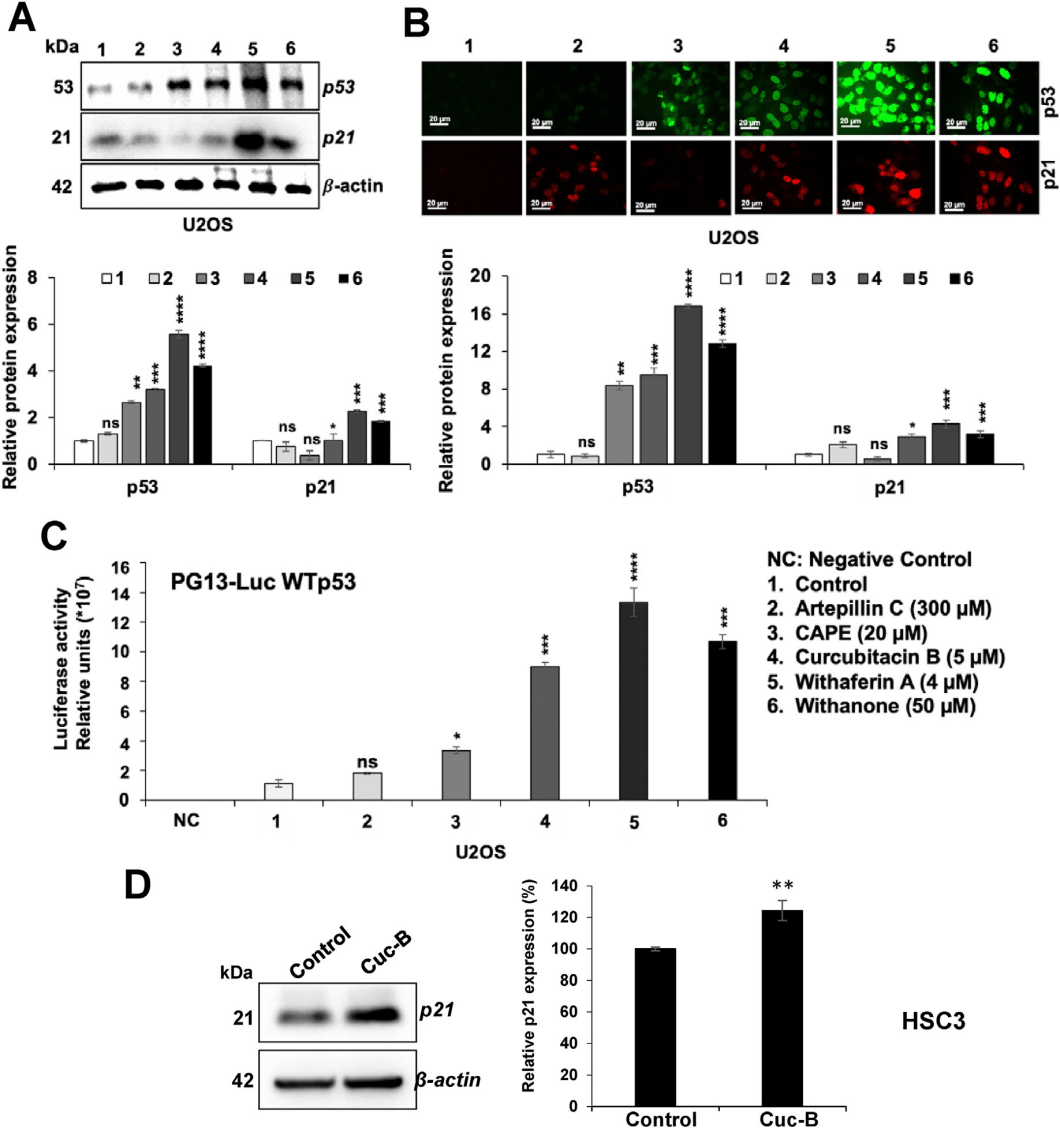
*In vitro* analysis of the comparative wild type p53 activation function of five natural compounds. A) Western blotting of control and treated U2OS cells for p53 and p21 proteins showed increase in the treated cells. Highest increase was observed in cells treated with Withaferin-A followed by Withanone, Cucurbitacin B, CAPE and Artepillin C. B) Immunocytostaining of control and treated U2OS cells showed increase in expression of p53 and p21 in the later. C) p53-dependent luciferase reporter assay in control and treated U2OS cells showed increase in wild type p53 activity on treated cells and was in accordance to the expression analysis. D) Western blotting of control and treated HSC3 cells (harboring p53^Ser46mutant^) for p21 showed its increase in the later. Blots (A and D) were probed with β-Actin as an internal loading positive control. The results from three independent experiments are shown with statistical analysis indicated as p values. p ​> ​0.05 (non-significant), p ​≤ ​0.05 (∗significant), p ​≤ ​0.01 (∗∗ very significant), p ≤ 0.001 (∗∗∗highly significant), and p ​≤ ​0.0001 (∗∗∗∗ extremely significant).

We next examined the effect of selected compounds on p53 activity *in vitro* using U2OS cells (possessing wild type p53) and HSC3 cells (possessing p53^Ser46mutant^). IC50 concentrations of each of the compounds were determined by cell viability assays and were consistent with the earlier reports (Dhanjal et al., 2021; Garg et al., 2020; A. Kaul et al., 2021; Kumar et al., 2022). Since wild type p53 function is well-related to its level of expression, U2OS cells treated with IC50 doses of each of the compounds (causing growth arrest as confirmed by viability assays; data not shown) were subjected to p53 expression analysis. As shown in Fig. 4A, cells treated with ARC, CAPE, Cuc-B, Wi-A or Wi-N showed increase in p53. Of note, Wi-A caused the highest increase followed by Wi-N, Cuc-B, CAPE and ARC (Fig. 4A). Consistent to the changes in p53, p21^WAF−1^ (a downstream effector of wild type p53 involved in growth arrest of cells) showed increase. Similar results were obtained by immunocytostaining (Fig. 4B). We also determined wild type p53 function by p53^WT^-dependent luciferase reporter assays. As shown in Fig. 4C, increase in wild type p53 function was recorded and it matched well with the level of expression. Furthermore, the computational analysis revealed that whereas Cuc-B, Wi-N and Wi-A could increase the binding of p53^S46PΔ^ to p62 yielding an increase in transcriptional activation function, CAPE and ARC were inert. Based on these analyses, Cuc-B was considered the strongest compound with multimodal p53-activating potential. We also examined the wild type transcriptional activation function of p53 in human oral squamous carcinoma that harbour p53^Ser46^ mutant. Since the mutant protein could not be detected on Western blots, we chose to determine the level of expression of p21^WAF1^, a reliable and consistent determinant of wild type p53 activity. Western blotting of control and Cuc-B-treated HSC3 cells revealed clear upregulation of p21^WAF1^endorsing reactivation of wild-type p53 function (Fig. 4D) and induction of growth arrest (Fig. 4E).

## 4 Discussion

It has been earlier reported that ARC, CAPE, Cuc-B, Wi-A and Wi-N cause growth arrest/apoptosis of cancer cells through multiple mechanisms of action (Bhargava et al., 2018; Garg et al., 2020; Malik et al., 2021; Narayanaswamy et al., 2014; Nigam et al., 2015; Radhakrishnan et al., 2021; Wadhwa et al., 2016) (Vaishnavi et al., 2012). One of these mechanisms is the abrogation of the mortalin-p53 complex that results in reactivation of p53 function and hence growth arrest/apoptosis/senescence in the cancer cells (Dundas et al., 2005; W. J. Lu et al., 2011b; Ma et al., 2006; Wadhwa et al., 1998). In another recent study, it was reported that the combination of Wi-A and CAPE possessed stronger anticancer activity and showed synergistic effect on reactivation of p53 function (Sari et al., 2020). It was shown that such synergism is due to the binding of Wi-A and CAPE to different sites of mortalin and p53 (Sari et al., 2020). Similarly, Cuc-B was shown to bind with p53 binding to Thr267 and Gly269 residues of mortalin involved in p53 interaction and could not form stable binding with residues (323–337) of p53 involved in mortalin interaction. On the other hand, Wi-N was shown to bind to residues of mortalin involved in p53 binding and residues of p53 involved in mortalin binding. Combination of Cuc-B and Wi-N, Cuc-B, and Wi-N was found to offer higher anticancer activity and effective at the low dose combination as well (Garg et al., 2020). In the earlier studies, it was reported that Wi-A, Wi-N, and CAPE could restore wild type p53 function in p53^Y220C^ mutant (Radhakrishnan et al., 2021; Sundar et al., 2019). In this mutant, absence of the hydrophobic aromatic ring of Tyr220 in DNA binding domain due to mutation creates a hydrophobic crevice. This crevice creates an instability makes the DNA binding domain unstable. The three molecules could bind to this druggable crevice caused by the mutation and stabilize the structure of p53.

In continuation with these studies, we currently examined the ability of these compounds to reactive wild type p53 function to p53^S46PΔ^ mutant. Computational analysis showed that there was a significant decrease in the binding energy of p53^S46PΔ^ and PH domain of p62 as compared to the p53^WT^-p62. The key interactions in the Lysine residues in the N-terminal of Phosphorylated p53 like Lys19 were decreased substantially which decreased the binding affinity of p53 with p62. We also found that there was a detachment of the p53 chain from the p62 causing the opening near the N-terminal p53, which may also attribute to the decrease in the binding energy. Cuc-B, Wi-N, and Wi-A showed stable binding with p53^S46PΔ^ and improved the binding of key Lysine residues (Lys19) and generated the structure that mimicked the phosphorylated p53. Such conformational change resulted in stronger interaction of p53^S46PΔ^ and PH domain of p62 as endorsed by increase in the MMGBSA binding energy between p53 and p62. Elongated string-like conformation increases the number of interactions formed by the residues in contact between p53 and p62 thereby its relevant in terms of increasing binding affinity between the two proteins.

The TAD domain of p53 have been known for its interaction with general transcription factors like TF11D and TF11H, co-activators, co-repressors (Okuda and Nishimura, 2014). In this study, the conformational changes and interaction of phosphorylated TAD2 domain binding with p62 were examined. The TF11H subunit p62 is a hub protein which plays important role in the transcription and DNA repair processes (Xiao et al., 1994). The p53-TAD2 domain exists in amphipathic alpha helical structure in unbound conformation. When bound with p62 and followed by phosphorylation at Ser46 and Thr55 residues the amphipathic alpha helical structure changes to an extended or elongated string like structure (Okuda and Nishimura, 2014). The conformational change is accompanied by increase in the binding energy due to the interaction of residues like Lys19, Gln97 of p62 and Trp53 of p53 which involved in a pocket engulfment and makes amino aromatic interaction. In the current study, Wi-N showed significant binding with Lys19 and Gln97 residues of p62 as well reversal of the small alpha-helical structure formed by the deficiency of phosphate in Ser46 residue into an elongated string-like conformation which is seen in wild type p53. While Cuc-B and Wi-N showed an increase in binding energy and significant interaction with residues Lys19 and Gln97 which is seen in wildtype p53. The clinical relevance of this increased binding affinity with p62- (a co transcription factor) could be predicted as wildtype p53 specific transcriptional activation function. In contrast to Cuc-B, Wi-N and Wi-A, the components from propolis (ARC and CAPE) failed to establish a stable interaction with p53^S46PΔ^, which was evident from the higher fluctuations observed in the RMSD plot (Fig. 1F). These data established the comparative anticancer effects of the five compounds. Whereas Wi-A, Wi-N and Cuc-B caused abrogation of mortalin-p53 interactions and mimicked the conformation generated by Ser46 phosphorylation, CAPE and ARC did not confer such effect. Further, Cuc-B could able block p53 and mortalin by binding into both of the p53 and mortalin interacting site which can act as a hindrance to p53-mortalin complex formation. Despite having strong binding energy ARC and CAPE do not interact within the p53-mortalin binding region after visualization. These data suggested that Wi-A, Wi-N and Cuc-B possess stronger and multimodal activity as compared to CAPE and ARC. This was indeed seen in the comparative *in vitro* assays on p53 expression and activity.

## 5 Conclusion

In this study, we have shown the ability of five natural compounds (ARC, CAPE, Cuc-B, Wi-A and Wi-N) in modulating wild type p53 function in cancer cells possessing either p53^WT^ or p53^S46PΔ^. Our results suggest that all the five compounds possess ability to disrupt mortalin-p53 interaction, although their strength varies. Wi-A, Wi-N and Cuc-B were defined as stronger inhibitors of these interactions as compared to CAPE and ARC. Furthermore, Wi-A, Wi-N and Cuc-B were able to bind to p53^S46PΔ^ and mimic the phosphorylated conformation in this mutant suggesting reactivation of wild type p53 function. The computational data was endorsed by *in vitro* cell based assays.

## CRediT authorship contribution statement

**Seyad Shefrin:** Conceptualization, Design, Formal analysis, Manuscript writing, Methodology, Investigation, Validation, Visualization. **Anissa Nofita Sari:** Contributed to experimental validation and manuscript writing. **Vipul Kumar:** Contributed to computational analysis. **Huayue Zhang:** Contributed to experimental validation. **Hazna Noor Meidinna:** Contributed to experimental validation. **Sunil C. Kaul:** Conceptualization, Manuscript writing and funding. **Renu Wadhwa:** Conceptualization, Design, Manuscript writing, Supervision, Project administration, Funding acquisition. **Durai Sundar:** Conceptualization, Design, Manuscript writing. Supervision, Project administration, Funding acquisition.

## Declaration of competing interest

The authors declare that they have no known competing financial interests or personal relationships that could have appeared to influence the work reported in this paper.
