# Supplementary material for: Comparative computational and experimental analyses of some natural small molecules to restore transcriptional activation function of p53 in cancer cells harbouring wild type and p53Ser46 mutant
Source: Curr Res Struct Biol. 2022 Sep 13;4:320–31. doi: 10.1016/j.crstbi.2022.09.002 (PMC9507986; doi:10.1016/j.crstbi.2022.09.002)

**Comparative computational and experimental analyses of some natural small molecules to restore transcriptional activation function of p53 in cancer cells harbouring wild type and p53^Ser46^ mutant**

Seyad Shefrin^1^, Anissa Nofita Sari^2^, Vipul Kumar^1^, Huayue Zhang^2^, Hazna Noor Meidinna^2^, Sunil C. Kaul^2^, and Renu Wadhwa^2,*^and Durai Sundar^1*^

Supplementary File-1

**Supplementary Figure 1: A)** Illustration representing the order of the sub-domains in human p53 protein. **B)** Hydrogen bonding plot of Wi-N, Wi-A, Cuc-B, and CAPE interactions with mortalin over 200ns simulation. **C)** Hydrogen bonding plot observed between p53 and p62 before and after Wi-N and Wi-A intervention in mutant Ser46 p53. **D)** Three-dimensional visualization of ARC interaction away from the p53 binding site of Mortalin. **E)** Three-dimensional visualization showing the interaction of ARC and Mortalin binding domain of p53. **F)** Root mean square deviation plot of ARC bound mortalin complex showing stable interaction. **G)** Root mean square deviation plot of ARC bound p53 complex showing stable interaction.


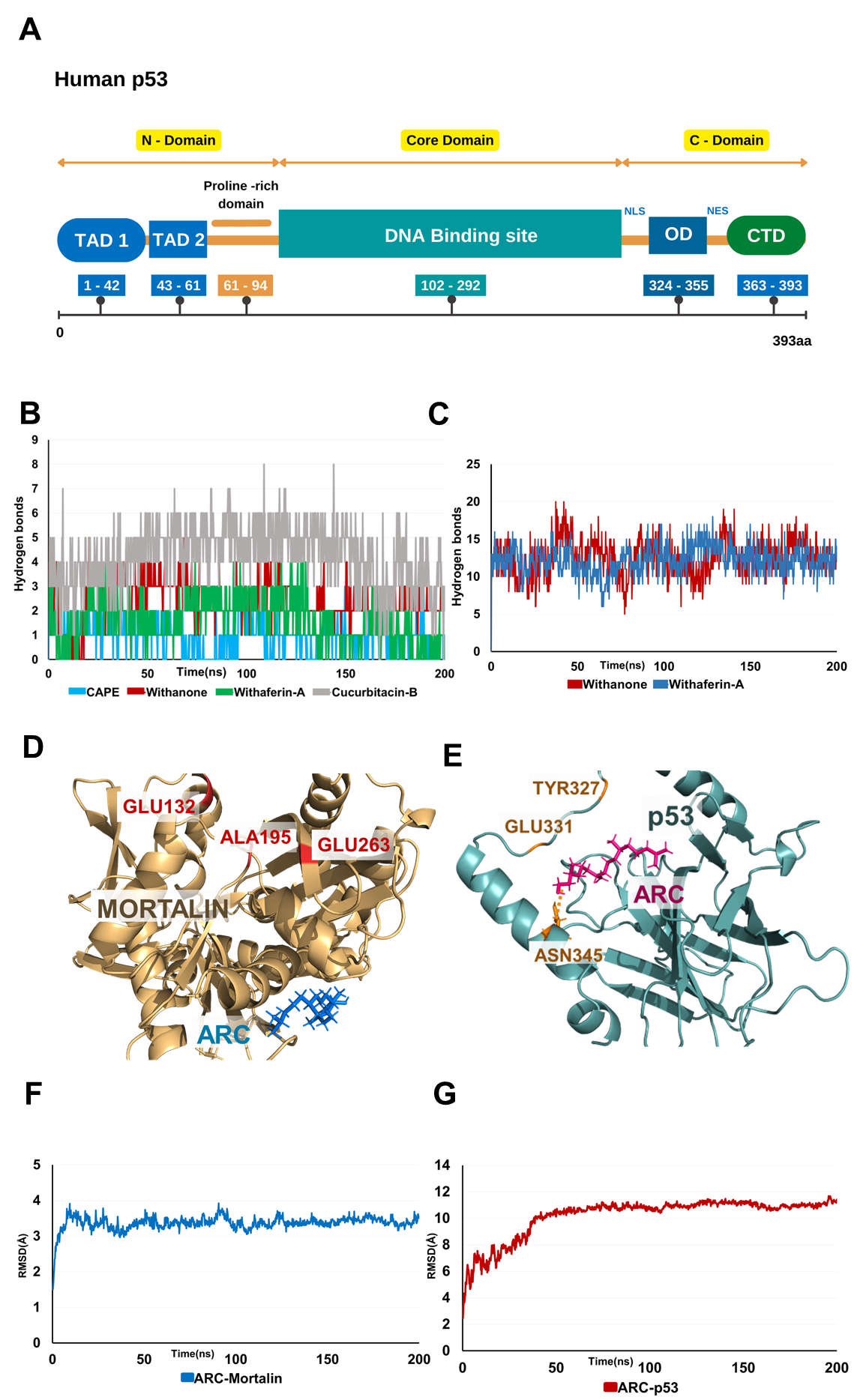


**Supplementary Figure 2: A)** Interaction fraction diagram of Wi-N with Mortalin over 200ns simulation. **B)** Interaction fraction diagram of CAPE with Mortalin over 200ns simulation. **C)** Interaction fraction diagram of Wi-A with Mortalin over 200ns simulation. **D)** Interaction fraction diagram of ARC with p53 over 200ns simulation. **E)** Frames extracted with an interval of 10ns from the simulation length 100-200ns showing the bending of Cuc-B which might be the reason for the fluctuation in the RMSD.


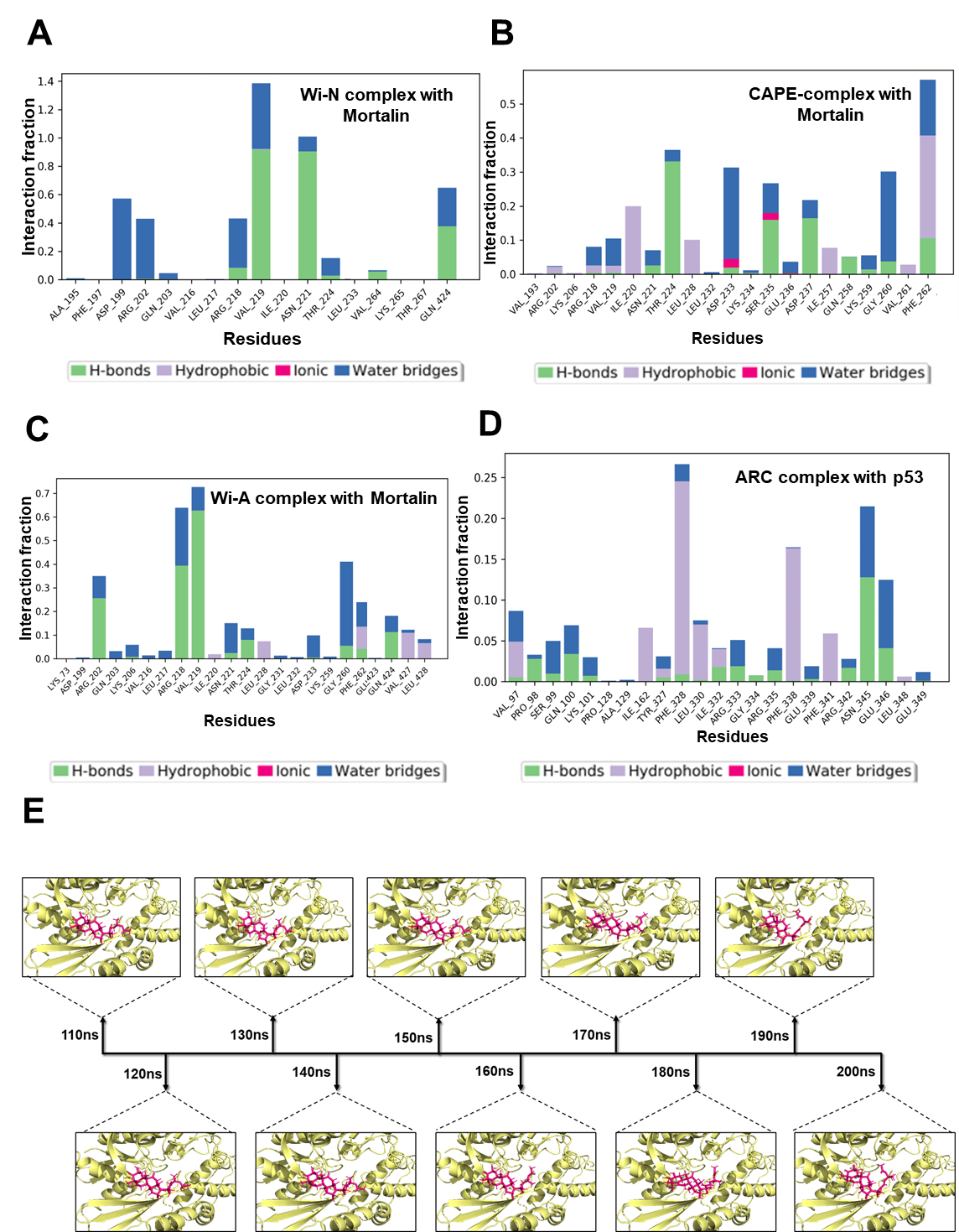

Supplement: Multimedia component 1 [file mmc1.docx]
